# Chronic Myeloid Leukemia in a Patient Receiving Tofacitinib: A Case Report and Literature Review

**DOI:** 10.1155/2019/2814504

**Published:** 2019-05-27

**Authors:** Georgio Medawar, Joseph Chahrouri, Rabih Said

**Affiliations:** ^1^Internal Medicine Department, St George Hospital University Medical Center and the University of Balamand, Beirut, Lebanon; ^2^Rheumatology Division, St George Hospital University Medical Center and the University of Balamand, Beirut, Lebanon; ^3^Hematology/Oncology Division, St George Hospital University Medical Center and the University of Balamand, Beirut, Lebanon

## Abstract

**Background:**

Tofacitinib is a new oral Janus kinase inhibitor that has shown promising clinical benefit in various rheumatologic diseases. However, many concerns related to the development of malignancies have been reported with its use.

**Case Presentation:**

A 43-year-old female patient received tofacitinib for refractory rheumatoid arthritis (RA). Two years after 5 mg bid daily dosing, the patient developed chronic myelogenous leukemia (CML), for which she received imatinib and tofacitinib was discontinued. She then remained in remission for rheumatoid arthritis and within the expected milestone outcome for her CML.

**Conclusion:**

This is the first reported case of CML after the use of tofacitinib. This event is of particular interest knowing the possible benefits tofacitinib carries in the treatment of CML demonstrated in a few studies.

## 1. Introduction

Tofacitinib is an oral Janus kinase (JAK) inhibitor that was approved in 2012 for moderate to severe active rheumatoid arthritis (RA), in 2017 for active psoriatic arthritis, and recently in 2018 for induction and maintenance of moderate to severe ulcerative colitis [1]. It primarily acts by inhibiting JAK3 and/or JAK1; subsequently, it leads to the inhibition of IL-15-induced phosphorylation of STAT5 and IL-6-induced phosphorylation of STAT1 and STAT3 [2].

The immune-modulator effect of tofacitinib in such a relatively new mechanism of action has raised a concern about adverse events, particularly malignancies [1, 3].

Herein, we report a case of a patient who developed CML after receiving tofacitinib for rheumatoid arthritis.

## 2. Case Presentation

A 43-year-old female patient with Hashimoto's thyroiditis was diagnosed with seropositive RA (anti-CCP 140, ref <20) in April 2013 when she presented with bilateral wrist, PIP, elbow, shoulder, knee, and ankle synovitis. She was started on prednisone taper at 20 mg/day and methotrexate 10 mg once a week optimized in increments up to 25 mg/week, with calcium, vitamin D, and folic acid supplements. Despite this therapy and despite significant improvement, there was recurrent, intermittent low-grade synovitis in both wrists. In mid-2016, X-rays revealed early erosions in the left ulnar styloid and capitate as well as the bases of the left second and third metacarpal bones. It was then decided, in January 2016, to start her on targeted therapy with tofacitinib 5 mg bid. The patient had rapid favorable response, and within 3 months, she was in clinical remission. The dose of methotrexate was progressively tapered down and stopped by the end of December 2016. The patient then remained in remission. Unfortunately, a routine laboratory test on June 23, 2016, showed leukocytosis of 27,500. The hemoglobin count was 11.3, and the platelet count was 610,000. A repeat CBC 5 days later confirmed leukocytosis with a WBC of 32,300 (7% metamyelocytes, 3% myelocytes, and 2% promyelocytes), hemoglobin count of 10.6, and platelet count of 703,000. Tofacitinib was discontinued, and the patient was referred to hematology/oncology.

Initial blood work was positive for t(9; 22) BCR-ABL (p210 b3a2) on FISH analysis. Bone marrow aspirate and biopsy showed myeloid and megakaryocytic hyperplasia with 2% blasts and a myeloid-to-erythroid ratio of 10 : 1. The karyotype showed t(9; 22) detected in all the examined cells. The flow cytometry showed that 5-6% of flow events express early myeloid markers.

The patient was diagnosed with chronic myelogenous leukemia (CML), and she was started on imatinib 400 mg. Two weeks later, blood tests showed a WBC of 18,200, platelet count of 514,000 and hemoglobin count of 9.9 that later further dropped to 6.5 with significant fatigue. Therefore, imatinib was decreased to 300 mg/day. Three months later, the patient remained in clinical remission for rheumatoid arthritis with hematological remission with WBC 4,700, Hg 12.5, and platelets 291,000. Her repeat quantitative PCR for BCR-ABL was 0.86%.

## 3. Discussion

Various malignancies have been reported after the use of tofacitinib in previous case reports and meta-analyses (Table 1). However, ongoing debate about the higher incidence rate of cancer after tofacitinib is not confirmed in the real-world, postmarketing treated population [12].

For instance, in the rheumatoid arthritis clinical development program, the incidence rate of malignancies in patients treated with tofacitinib, excluding nonmelanoma skin cancer (NMSC), was reported to be 0.85/100 patient-year (py) (107 out of 5671 patients) in a total tofacitinib exposure of 12,644 py. These include both solid cancers (lung, breast, and gastric cancer) and hematologic malignancies (lymphoma and T-cell CLL) [3]. Cohen et al. present a more extended analysis with 6,194 patients included and a total exposure of 19,406 py (over 8.5 years). The incidence rate of malignancies was 0.89/100 py, and they included lung disorders, breast disorders, and lymphoma/lymphoproliferative disorders [4]. In both analyses, there was no association between the dose of tofacitinib and the risk of malignancy (except for NMSC), and the malignancy rate remained relatively stable by 6-month intervals. Similarly, a Japanese long-term extension (LTE) study reported an incidence rate of 1.2/100 py of malignancies (19 out of 486 patients) that include gastric, breast, ovarian, colon, lung, fallopian tube, thyroid, and esophageal cancer, in addition to acute myeloid leukemia (AML), lymphoproliferative disorders, liposarcoma, and transitional cell carcinoma [5]. However, a large meta-analysis showed that there is no statistically significant increase in any type of malignancies with the use of tofacitinib in patients with RA [12]. Furthermore, the risk of NMSC, lung cancer, and lymphoma/lymphoproliferative disorders was previously reported to be increased in patients with rheumatoid arthritis [13, 14]; therefore, the analysis of cancer development in this patient population would be challenging.

On the contrary, the data on cancer development in psoriatic arthritis patients treated with tofacitinib are more unclear. For instance, two out of three phase 3 clinical trials reported malignancies as adverse events. In OPAL Broaden, 3 patients developed solid tumors (excluding NMSC) out of 107 patients (2.8%) taking continuous 5 mg bid tofacitinib for up to 12 months [6]. In the ongoing OPAL Balance trial, after 24 months of follow-up, 2 cases of malignancies were reported among the 680 enrolled patients (0.3%) [7], whereas in OPAL Beyond, no malignancies were reported [8].

Likewise, in ulcerative colitis, three phase 3 clinical trials (OCTAVE Induction 1, OCTAVE Induction 2, and OCTAVE Sustain) evaluated the safety and efficacy of tofacitinib in induction and maintenance. No malignancies (excluding NMSC) were reported in any of these trials, but an ongoing LTE study revealed 8 cases giving an overall incidence rate of 0.48/100 py (combining OCTAVE trials with the LTE study) [9, 10].

In addition, Xie and Ma reported a case of AML 2 years after the use of tofacitinib for refractory ulcerative colitis [11], despite a possible increased risk of AML with ulcerative colitis [15].

Natural killer (NK) cells, in addition to other cytokine transduction signaling (such as interferon types I and II), have been shown to have a major inhibitory role in tumorigenesis [16]. Tofacitinib has been shown to decrease the number of NK cells as well as the signaling transduction of various cytokines [2, 17]. This might hypothetically be a mechanism by which tofacitinib can increase the risk of some malignancies.

From another perspective, various studies have investigated the role of the JAK/STAT pathway in the pathophysiology of CML [18, 19]; BCR-ABL activates several signaling pathways such as PI3K, RAF, ERK, MEK, and JAK. The latter transduces signals via STAT and plays essential roles in cell proliferation and differentiation in leukemia [18]. In addition, few in vitro studies have shown that the use of JAK inhibitors with or without imatinib on CML cell lines had a modest positive outcome with JAK2/JAK3 inhibitors alone and a marked effect when combined (synergic effect). This suggests that targeting JAK may carry a potential therapeutic option for CML [18–21]. These findings led to the integration of JAK inhibitors in recent recruiting/ongoing clinical trials for treatment of CML (see Web References [22] and [23]).

To our knowledge, this is the first reported case of CML that occurred 2 years after tofacitinib use at 5 mg bid in a patient with rheumatoid arthritis. It is difficult to tell whether our patient developed a de novo CML or she already had acquired the BCR-ABL translocation during her treatment for rheumatoid arthritis which was possibly “silenced” by inhibiting the JAK3 pathway with tofacitinib. Moreover, a solid cause-effect relationship between the use of tofacitinib and the development of CML cannot be established. However, by discontinuing tofacitinib and treating her with imatinib, the patient has been so far in remission for her rheumatoid arthritis and within the expected milestone outcome for her CML.

Whether imatinib contributed to the maintenance of her rheumatoid arthritis in remission or not is not known. An extensive mutational workup and studying the effect of reintroduction of tofacitinib in case of relapse of either rheumatoid arthritis or CML would be interesting to perform in this case.

## 4. Conclusion

Despite the potential benefits of tofacitinib in the treatment of CML, we illustrated a unique case of CML that developed after its use for RA. It may represent a potential side effect that may be added to the list of malignancies associated with this drug. In contrast, the response of both RA and CML to imatinib shed the light on the interrelation of pathways between RA and CML and may be the subject of many interesting studies.

## Figures and Tables

**Table 1:** Reported cases of malignancies with the use of tofacitinib.

	Reported malignancies (excluding NMSC)	Malignancy crude IR	Malignancy IR/100 py
*Rheumatoid arthritis*			
Curtis et al. [3]	Lung, breast, and gastric lymphoma and T-cell CLL	1.88	0.85
Cohen et al. [4]	Lung disorders, breast disorders, and lymphoma/lymphoproliferative disorders	2.79	0.89
Yamanaka et al. [5]	Gastric, breast, ovarian, colon, lung, fallopian tube, thyroid, esophageal, and lymphoproliferative disorders, acute myeloid leukemia, liposarcoma, and transitional cell carcinoma	3.9	1.2
*Psoriatic arthritis*			
OPAL trials [6–8]^*∗*^	Breast carcinoma, transitional cell carcinoma, and vulvar squamous cell carcinoma	2.8 (OPAL Broaden)	2.8 (OPAL Broaden)
		0.3 (OPAL Balance)	— (OPAL Balance)
*Ulcerative colitis*			
OCTAVE trials + LTE [9, 10]^*∗∗*^	—	0.48	—
Xie and Ma [11]	Acute myeloid leukemia	—	—

^*∗*^The cancer types mentioned were only reported in OPAL Broaden. In the ongoing OPAL Balance, there was no specification of the cancer types. ^*∗∗*^In the ongoing LTE study (NCT014770612), there was no specification of the cancer types.

## Abbreviations

JAK: Janus kinase
RA: Rheumatoid arthritis
CML: Chronic myelogenous leukemia
AML: Acute myelogenous leukemia
NMSC: Nonmelanoma skin cancer
LTE: Long-term extension.

## Additional Points

*Clinical Practice.* Many solid and hematologic malignancies have been reported after the use of tofacitinib. Our first-time reported case of CML is of special interest: (i) It is the first malignancy type that has a direct pathway relation with the drug's targets. (ii) The drug itself has potential benefits in treatment of CML. (iii) We are not sure if the drug delayed or potentiated the development of CML. (iv) Treatment of CML with imatinib led to good response to both RA and CML. These observations raise many questions that can be addressed in the future: (i) What is the mutational status of patients receiving JAK3 inhibitors who develop CML? (ii) Do JAK inhibitors really work in CML? (iii) What is the effect of reintroducing JAK3 inhibitors in patients with relapsed CML who previously received JAK inhibitors? (iv) Does imatinib play a role in rheumatoid arthritis?
